# Identifying research priorities for pituitary adenoma surgery: an international Delphi consensus statement

**DOI:** 10.1007/s11102-025-01502-7

**Published:** 2025-03-05

**Authors:** Nicola Newall, Alexandra Valetopoulou, Danyal Z. Khan, Anouk Borg, Pierre M. G. Bouloux, Fion Bremner, Michael Buchfelder, Simon Cudlip, Neil Dorward, William M. Drake, Juan C. Fernandez-Miranda, Maria Fleseriu, Mathew Geltzeiler, Joy Ginn, Mark Gurnell, Steve Harris, Zane Jaunmuktane, Márta Korbonits, Michael Kosmin, Olympia Koulouri, Hugo Layard Horsfall, Adam N. Mamelak, Richard Mannion, Pat McBride, Ann I. McCormack, Shlomo Melmed, Katherine A. Miszkiel, Gerald Raverot, Thomas Santarius, Theodore H. Schwartz, Inma Serrano, Gabriel Zada, Stephanie E. Baldeweg, Angelos G. Kolias, Hani J. Marcus, the PitCop Collaborative

**Affiliations:** 1 National Hospital for Neurology and Neurosurgery, London, UK; 2 Department of Computer Sciences, UCL Hawkes Institute, University College London, London, UK; 3 Division of Medicine, Department of Experimental and Translational Medicine, University College London, London, UK; 4 University Hospital Erlangen, Erlangen, Germany; 5 Oxford University Hospitals NHS Foundation Trust, Oxford, UK; 6 Barts and the London School of Medicine, Queen Mary University of London, London, UK; 7 Stanford University School of Medicine, 213 Quarry Road, Palo Alto, USA; 8 Oregon Health & Science University, Portland, USA; 9 The Pituitary Foundation, Bristol, UK; 10 Cambridge University Hospitals NHS Foundation Trust, Cambridge, UK; 11 The Francis Crick Institute, 1 Midland Road, London, NW1 1AT UK; 12 Cedars-Sinai Medical Center, Los Angeles, CA USA; 13 St Vincent’s Hospital Sydney, Darlinghurst, NSW Australia; 14 Department of Endocrinology, French Reference Center for Rare Pituitary Diseases HYPO, Hospices Civils de Lyon, 69002 Lyon, France; 15 Keck School of Medicine, University of Southern California, Los Angeles, CA USA; 16 NY, USA; 17 Department of Oncology, University College London Hospitals NHS Foundation Trust, London, UK; 18 University of Cambridge, Cambridge, UK; 19 Department of Endocrinology, University College London Hospitals NHS Foundation Trust, London, UK

**Keywords:** Pituitary surgery, Research priorities, Priority setting

## Abstract

**Purpose:**

Pituitary surgery is the mainstay treatment for most pituitary adenomas, but many questions remain about perioperative and long-term management and outcomes. This study aimed to identify the most pressing research priorities in pituitary surgery with input from patients, caregivers, and healthcare professionals.

**Methods:**

An initial survey of patients, caregivers, and healthcare professionals assembled priorities related to preoperative care, surgical techniques, and postoperative management in pituitary surgery. Priorities were thematically grouped into summary priorities, and those answered by existing evidence were omitted following a literature review. An interim survey asked patients, caregivers, and healthcare professionals to select their top 10 priorities from the remaining list. The highest-ranked priorities advanced to a consensus meeting, where the top 10 questions were prioritized.

**Results:**

In the initial survey, 147 participants—60.5% of whom were patients, caregivers, or patient support group representatives—submitted 785 priorities, which were then condensed into 52 summary priorities.

After a literature review, 33 unanswered priorities were included in the interim survey, completed by 155 respondents, of whom 54.2% were patients, caregivers, or patient support group representatives. The top-ranked priorities were discussed by 14 participants (7 patients and 7 healthcare professionals) during a consensus meeting. The top 10 priorities covered a variety of themes including enhancing diagnosis and management of pituitary adenomas, advancing surgical techniques and technologies, optimizing the prediction of outcomes and complications, and improving patient support and follow-up.

**Conclusions:**

The top 10 research priorities in pituitary surgery aim to align researchers and direct funding in order to maximize impact and champion patient representation.

**Supplementary Information:**

The online version contains supplementary material available at 10.1007/s11102-025-01502-7.

## Introduction

Pituitary adenomas are common benign tumors, accounting for around 15% of all intracranial neoplasms [1]. Their detection within the general population is increasing, affecting 76 to 116 cases per 100,000 [2]. Aside from the scenario of an incidental pituitary adenoma, the typical presentation includes visual deterioration for macroadenomas and/ or clinical features of hormonal imbalances. Management is guided by several factors, mainly by hormonal hypersecretion and adenoma size, typically involving a multidisciplinary approach [1].

Surgical resection, via an endonasal transsphenoidal approach, is the mainstay of treatment for symptomatic non-functioning pituitary adenomas (NFPA), most functioning adenomas and asymptomatic patients whose tumour was found by chance, but with anatomical features mandating a preventive approach (e.g., incidental adenoma compressing the chiasm) [3–7]. Over recent years, there have been significant advances in pituitary adenoma surgical techniques and technology. Despite these advances, there remain challenges and unmet needs for patients undergoing pituitary adenoma surgery. For example, early complications such as post-operative cerebrospinal fluid (CSF) rhinorrhea and dysnatraemia remain common, in up to 5% and 30% of cases respectively in some centres [8, 9]. Similarly, failure to achieve remission for hypersecreting adenomas and recurrence remain common, and efforts to improve these rates have plateaued in recent years [10]. Furthermore, there is an increasing appreciation of the adverse impact of pituitary adenomas across several quality of life (QoL) domains relating to both physical and mental health measures [11]. This impact on QoL persists in patients despite ‘good’ outcomes by conventional metrics, with the exact reasons for this misalignment unclear.

To address these challenges, research should ideally be focused on agreed priority areas, aligned to patient perspectives [12]. Current clinical research is largely reflective of the interests of researchers in academia or industry [13]. This has therefore led to a mismatch in research priorities for patients, clinicians, and researchers and at times to inefficient and ineffective research reports [14].

Priority setting studies aim to consolidate research priorities through consensus for areas of healthcare where there are substantial gaps in research. Research priority studies bring patients, caregivers, and healthcare professionals together to identify and prioritize the research that matters most to them, aligning both patients’ interests and researchers’ objectives. To date, there have been several research priority studies covering a variety of neurosurgical disease areas including degenerative cervical myelopathy, brain and spine cavernous malformations, and spinal cord injury [15–17]. However, there has been no study focusing on the research priorities for pituitary surgery.

This study aims to identify the most important research priorities in pituitary surgery from the joint perspective of patients, caregivers, and clinicians. The findings will inform healthcare researchers and funding agencies, helping to shape the direction of future research and resource allocation [18].

## Methods

### Overview

**Fig. 1:**
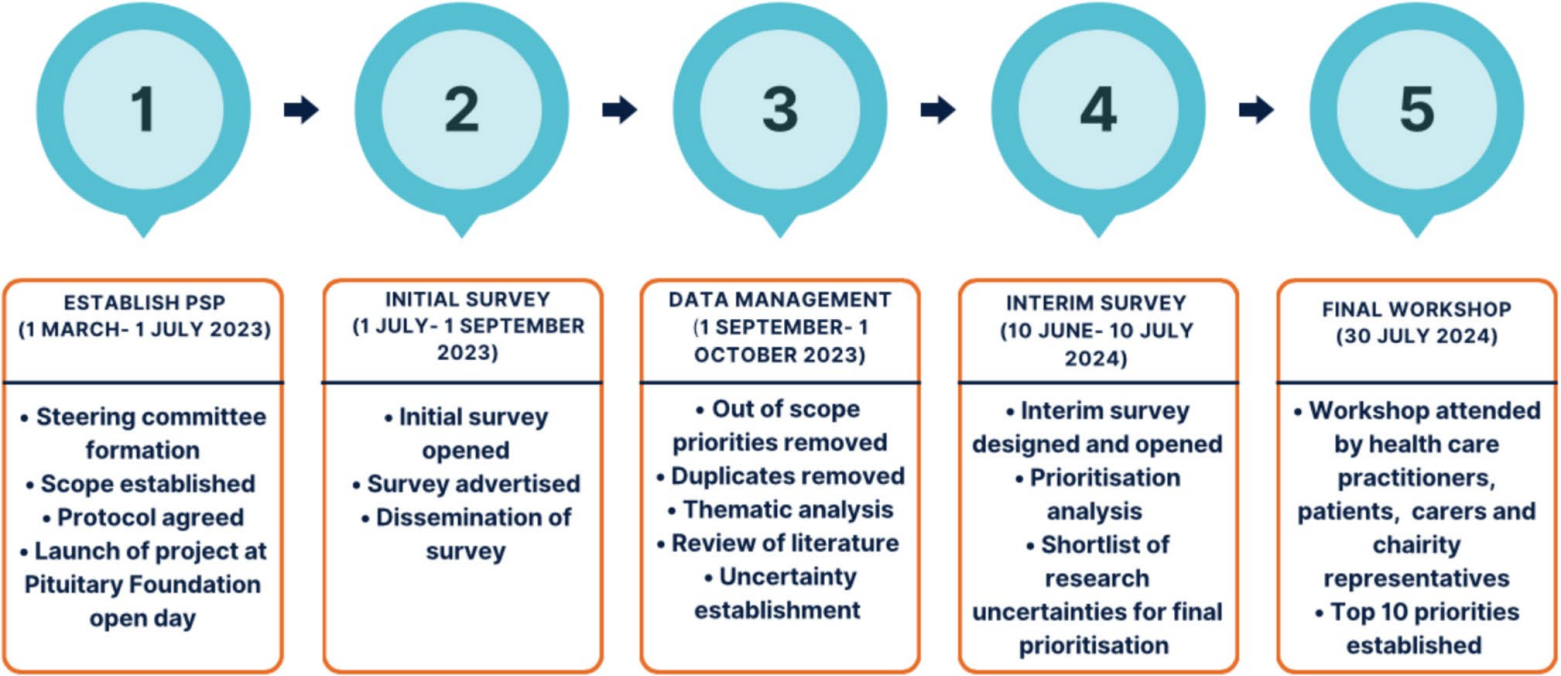
Priority setting partnership (PSP) study timeline

An international multistakeholder research priority consensus study designed by an expert steering committee, comprised two Delphi surveys and a consensus workshop (Fig. 1). The first survey aimed to gather a comprehensive list of priorities, while the interim survey and consensus workshop aimed to iteratively refine the long list into the most pressing research priorities. Study design and report generation were guided by the REPRISE guidelines [19]. Ethics approval was granted by the University of Cambridge Psychology Research Ethics Committee (PRE.2023.080).

### Scope

The scope of the research priority study encompassed all areas of pituitary adenoma surgery research, including both functioning and NFPAs undergoing surgical resection.

Decisions on whether proposed submissions were in- or out-of-scope were made by the steering group. Out-of-scope priorities were considered those not related to pituitary adenoma surgery or those that were too broad.

### Study management

This collaborative study included multiple stakeholders throughout—in the steering committee, management committee, and participants. Stakeholders included healthcare professionals involved in the diagnosis and surgical management of pituitary adenomas, such as endocrinologists, neurosurgeons, ophthalmologists, oncologists, otolaryngologists, radiologists, and clinical nurse specialists. Service users, including patients, caregivers, and family members, along with charity representatives from the Pituitary Foundation, were also involved.

A steering group was formed with experts representing each stakeholder group and held overall responsibility for the study, including study design, consensus generation, and dissemination of results. The steering group consisted of representatives from both UK and international professional and charitable organizations, including the Pituitary Society, the Pituitary Foundation (an international charity for patients with disorders of the pituitary gland), the Society for Endocrinology, and the European Society of Ophthalmology. The steering group consisted of 12 neurosurgeons, 10 endocrinologists, 1 pathologist, 1 ophthalmologist, 1 oncologist, 1 otolaryngologist, 1 radiologist, 3 neurosurgical trainees, 1 clinical nurse specialist, and 3 service users with lived experience, all of whom were charity representatives from the Pituitary Foundation (Appendix 1).

A management group oversaw the day-to-day administration of the priority study. The management group consisted of six healthcare professionals: two pituitary neurosurgeons, one endocrinologist and three neurosurgical trainees.

### Initial survey

The initial survey was created by the steering group to gather potential research priorities as well as the demographics of the respondents including age, biological sex, location, and stakeholder group. The survey asked the following four questions to structure stakeholder feedback:

- What question(s) about the diagnosis of pituitary adenomas requiring surgery would you like to see answered by research?
- What question(s) about the surgical treatment of pituitary adenoma would you like to see answered by research?
- What question(s) about the long-term care and follow-up after pituitary adenoma surgery would you like to see answered by research?
- What other question(s) about pituitary adenoma surgery that do not fit into the above categories would you like to see answered by research?

A dedicated webpage (www.pit-cop.com) was created for the study, providing information resources, promotional videos (Supplementary Video 1) and direct links to the survey, which was conducted via Qualtrics. The survey was launched at the 2023 annual Pituitary Foundation Open Day meeting. The survey was open from 1st July 2023 to 1st September 2023 to patients, their families and caregivers, and healthcare professionals involved in the diagnosis and treatment of pituitary adenomas. The survey targeted patients, healthcare professionals, and researchers, ensuring comprehensive outreach to key stakeholders. The survey was promoted and disseminated through the Pituitary Foundation, the Society for Endocrinology, and the Pituitary Society. Furthermore, it was promoted via the study’s official social media platform (X: @PitCop2023) and through the Steering Committee’s professional network, leveraging email communications and various social media channels to maximize outreach. Promotional materials supported by our local public engagement team and the Pituitary Foundation were used to support recruitment and study understanding. All responses were anonymized and there was no limit on the number of research priorities that could be submitted. Responses were collected until data saturation, defined as the point when steering group members agreed that no new priorities or themes were emerging.

An information specialist (N.N.) reviewed the initial survey responses, removed duplicates, excluded out-of-scope submissions with steering group consensus, and grouped the remaining research priorities into themes. In-scope priorities were cross-checked against the literature for sufficient evidence, and summary priorities were generated from unanswered priorities for the interim survey.

### Interim survey

The interim survey aimed to rank unanswered research priorities from the initial survey by asking stakeholders to select their top 10. In-scope unanswered priorities identified in the initial survey were digitized and presented via Qualtrics, with the survey running from 10th June to 10th July 2024. Dissemination followed the same approach as the initial survey, with links shared through relevant organizations and sent directly to those who participated in the initial survey. Responses were analyzed by stakeholder group, and the most frequently ranked priorities were brought forward to the final research priority setting workshop.

### Consensus meeting

The meeting aimed to establish the top 10 research priorities from the shortlist of priorities via an online consensus workshop. The final prioritization workshop included 14 representatives, with equal representation of healthcare professionals and service users (1:1 ratio). Representatives were recruited from the steering committee and from the steering committees’ network. The meeting was held online via Zoom® on 30th July 2024.

## Results

### Initial survey

A total of 147 responses were received (88 patients, 1 relative and caregiver, 57 healthcare professionals, and 1 who preferred not to say), across 14 countries (Table 1).

**Table 1:** Demographics of survey participants

	Initial survey (n = 147) (%)	Interim priority setting survey (n = 155) (%)
*Stakeholder sub-group*		
Healthcare professional	57 (38.7)	71 (45.8)
Neuroendocrinologist	21 (36.8)	27 (38.0)
Neurosurgeon	29 (50.9)	37 (52.1)
Oncologist	1 (1.8)	1 (1.4)
Ophthalmologist	2 (3.5)	2 (2.8)
Otolaryngologist	1 (1.8)	1 (1.4)
Pathologist	1 (1.8)	1 (1.4)
Radiologist	1 (1.8)	1 (1.4)
Specialist Nurse	1 (1.8)	1 (1.4)
*Patients/caregivers/patient representatives*	89 (60.5)	84 (54.2)
Patient	88 (98.9)	80 (51.6)
Caregiver	1 (1.1)	2 (1.3)
Charity representative	0	2 (1.3)
Prefer not to say	1 (0.7)	0
*Geographical region*		
Europe	122 (82.1)	120 (77.4)
North America	15 (10.2)	20 (13.0)
Oceania	5 (3.4)	3 (1.94)
Asia	4 (2.7)	4 (2.56)
Africa	3 (2.0)	4 (2.6)
South America	1 (0.7)	1 (0.7
*Ethnicity*		
White	113 (90.5)	134 (86.5)
Asian or Asian British	8 (5.4)	12 (7.7)
Black, Black British, Caribbean or African	5 (3.5)	5 (3.2)
Any other ethnic group	1 (0.7)	1 (0.6)
Prefer not to say	0	3 (1.9)
*Age of respondents*		
< 30	4 (2.7)	10 (6.5)
31–50	58 (39.5)	61 (39.4)
51–70	69 (46.9)	67 (43.2)
71 +	16 (10.9)	17 (11.0)
*Sex*		
Female	73 (49.7)	79 (51.0)
Male	74 (50.3)	76 (49.0)

Respondents submitted a total of 785 unique research priorities. After removal of out-of-scope submissions, 708 remained. A total of 52 research priorities were reviewed by the steering group and consolidated to create 33 refined research priorities. No priorities were found to be sufficiently answered by existing research during evidence checking, and therefore, all were progressed to the interim survey (Supplementary Table 1).

### Interim survey

Respondents were asked to pick their top 10 research priorities from a list of 33. A total of 155 respondents from 17 countries completed the survey online, comprising 80 patients (51.6%), 2 caregivers (1.3%), 2 charity representatives (1.3%), and 71 healthcare professionals (45.8%) (Table 1).

The top-ranked priorities in the interim survey were different between patients, caregivers and charity representatives, and healthcare professionals. Patients, caregivers, and charity representatives prioritised questions focused on improving long-term outcomes, QoL, and communication. Healthcare professionals prioritised questions aimed at refining diagnosis and improving outcomes through innovative techniques. Full details of responses by the respondent groups can be found in Supplementary Table 1. There were no notable differences in the prioritization of questions across geographic regions. However, the small number of participants from certain regions limits the ability to draw definitive conclusions about potential regional variations.

Among the top 10 priorities selected by both groups—patients, caregivers and charity representatives, and healthcare professionals—three priorities overlapped (Supplementary Table 1). To maintain balance for the final workshop, the top 21 priorities were shortlisted, incorporating the top 10 priorities from each group. The combined list is shown in Supplementary Table 2.

### Final consensus meeting

The final meeting included 14 participants: 7 patients (2 of whom were charity representatives), 3 consultant neurosurgeons, 3 consultant endocrinologists, and a consultant ophthalmologist. The day was co-ordinated and run by three steering group members (HJM, NN, DZK). Throughout the workshop, each person was asked in turn to share their views, allowing for inclusive participation. Before the workshop, delegates were asked to rank the short list of 21 priority questions from 1 to 21 (Supplementary Table 2). This served as a personal reference for each participant to guide their views during the workshop.

**Fig. 2:**
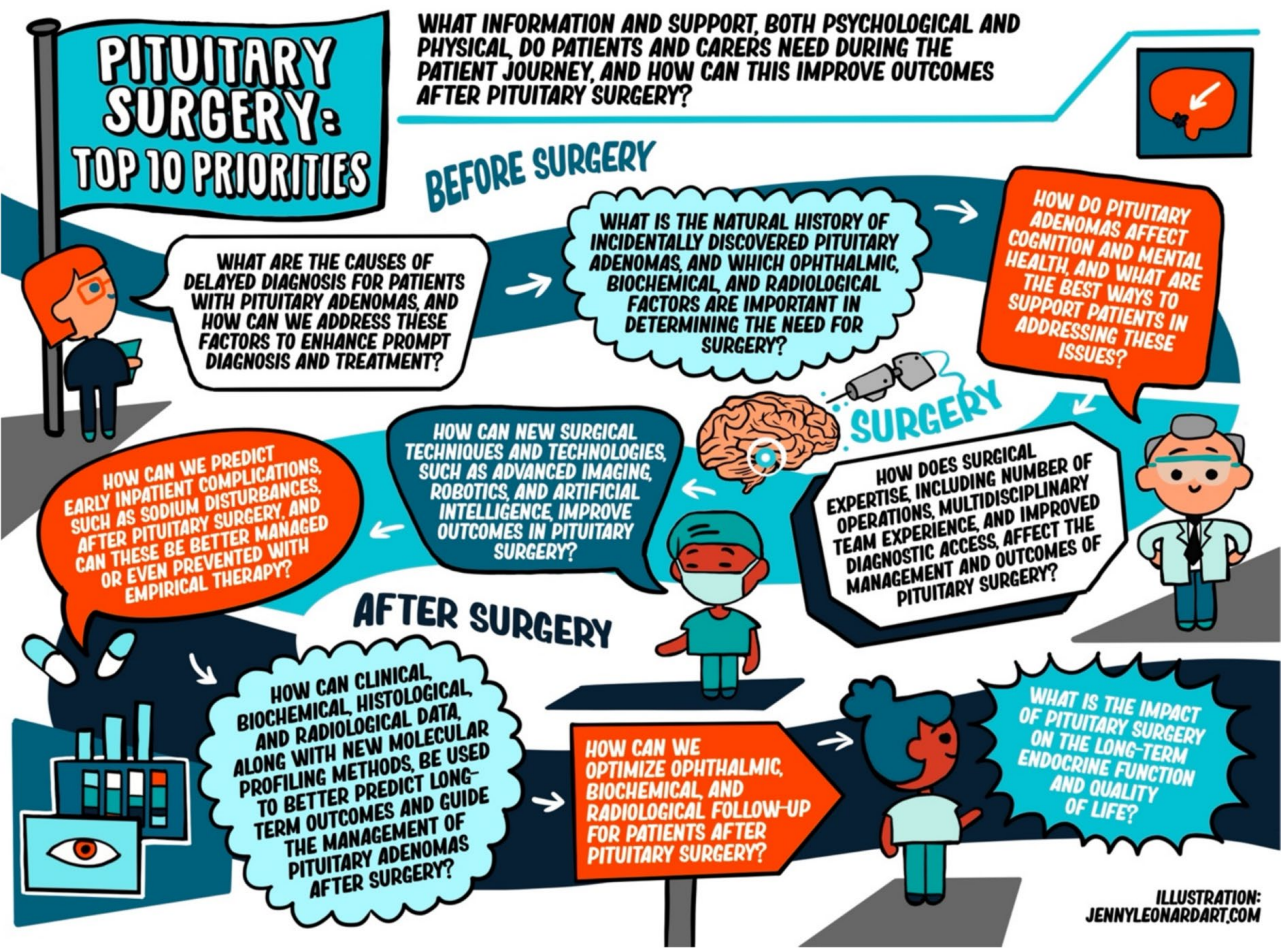
Infographic of the top 10 research priorities in pituitary surgery

Prioritisation was undertaken in three sessions. Session 1 involved all delegates providing a rank order of 1–3 from their top 3 and bottom 3 priorities. These priorities were then separated into 3 lists: (1) highest-ranked priorities (2) priorities not mentioned (3) lowest-ranked priorities. Session 2 featured a detailed review of each of the aforementioned lists, exploring divergent views and justifying different perspectives. This discussion recommended combining 5 priorities due to their overlap (Supplementary Table 2). This resulted in the production of 15 priorities. Session 3 focused on establishing the top 10. Following recommendations, priorities were iterated, and the revised top 10 was circulated to workshop attendees for final ranking via an online survey. The final top 10 research priorities are detailed in Table 2, illustrated in Fig. 2, and supported by Supplementary Video 2.

**Table 2:** Top 10 ranked research priorities in pituitary surgery

Rank	Top 10 priorities
1	What is the impact of pituitary surgery on the long-term endocrine function and quality of life?
2	How can clinical, biochemical, histological, and radiological data, along with new molecular profiling methods, be used to better predict long-term outcomes and guide the management of pituitary adenomas after surgery?
3	What are the causes of delayed diagnosis for patients with pituitary adenomas, and how can we address these factors to enhance prompt diagnosis and treatment?
4	How can new surgical techniques and technologies, such as advanced imaging, robotics, and artificial intelligence, improve outcomes in pituitary surgery?
5	What information and support, both psychological and physical, do patients and carers need during the patient journey, and can this improve outcomes after pituitary surgery?
6	How does surgical expertise, including number of operations, multidisciplinary team experience, and improved diagnostic access, affect the management and outcomes of pituitary surgery?
7	How do pituitary adenomas affect cognition and mental health, and what are the best ways to support patients in addressing these issues?
8	What is the natural history of incidentally discovered pituitary adenomas, and which ophthalmic, biochemical, and radiological factors are important in determining the need for surgery?
9	How can we predict early inpatient complications, such as sodium disturbances, after pituitary surgery, and can these be better managed or even prevented with empirical therapy?
10	How can we optimize ophthalmic, biochemical, and radiological follow-up for patients after pituitary surgery?

## Discussion

Pituitary surgery is critical for the management of pituitary nonfunctioning macroadenomas or secreting adenomas for most patients. Through international multistakeholder consensus, this PSP has identified the ‘top 10 priorities’ for future pituitary surgery research. The PSP collaborated with a variety of patient networks and professional bodies throughout the process to incorporate diverse perspectives and expertise in addressing the key priorities in pituitary surgery. Together, these established priorities provide a resource to inform research funding bodies and guide future pituitary surgery research. To our knowledge, this is the first attempt to formally identify research priorities in pituitary surgery.

### Principal findings

The key themes emerging from the PSP include enhancing diagnosis and management, advancing surgical techniques and technologies, improving patient support and follow-up, and optimizing the prediction of outcomes and complications.

Improving the diagnosis and management of pituitary adenomas requires addressing delays in diagnosis, identified as the 3rd most important research priority. The often incidental, insidious, and nonspecific nature of these tumors makes them particularly challenging to diagnose, leading to significant under-recognition by healthcare professionals. On average, patients with NFPAs face a 2–3-year diagnostic delay, those with Cushing’s disease experience a 3–4-year delay, and those with acromegaly encounter a 5.5-year delay from symptom onset [20, 21]. Left undiagnosed or untreated, NFPAs and functioning adenomas can result in progressive tumor growth and invasion, further complicating treatment and resulting in irreversible morbidity and mortality [5, 22]. Thus, timely diagnosis is essential to maximize the chances of cure and reduce the systemic morbidity and mortality. To overcome these diagnostic challenges, it is essential to raise awareness at all levels of healthcare, enhance early detection efforts, improve understanding of the natural course of incidental adenomas, and ensure timely treatment. Emerging technologies, including computer-aided tools like natural language processing (NLP), are being explored to aid in early detection by identifying patterns in medical data, offering new avenues for earlier intervention [9].

Despite many advances in modern pituitary surgery [3–7], challenges persist in identifying the tumor-gland interface and safely resecting large or laterally extending tumors, while protecting surrounding neurovascular structures. Even when tumors are visualized, achieving the necessary dexterity and reach with current endoscopic instruments can be difficult, potentially affecting the extent of resection and overall surgical outcomes. As reflected by priority 4, there is a critical need to advance surgical techniques and integrate new technologies to overcome these challenges. Recent innovations in surgical technology, including advanced imaging, robotic systems, and artificial intelligence (AI) offer promising solutions. Studies have shown that AI-driven real-time instrument tracking in endoscopic pituitary surgery can predict a surgeon’s skill level, offering valuable insights into their performance. Additionally, an AI-assisted video coaching program has led to improvements in surgical performance and outcomes, highlighting the emerging impact of AI technologies in advancing surgical training and improving patient outcomes [23, 24]. Newer imaging modalities, including 7-Tesla MRI and molecular PET imaging integrated with machine learning, may improve lesion detection and tumor delineation, optimizing pre-operative planning [25–28]. Intraoperative imaging techniques, including MRI, ultrasound, and fluorescence imaging can further aid in real-time tumor identification and accurate delineation of the tumor-normal gland interface, enhancing surgical precision thereby reducing post-operative complications [29–32]. Additionally, the use of intra-operative robotic systems offers added articulation and improved precision during resection, increasing dexterity in addressing challenging surgical areas. The integration of robotic platforms—such as teleoperated, shared-control, and handheld systems with articulated end effectors—has shown promising results in pre-clinical studies and holds the potential to significantly transform pituitary surgery [33–35]. For instance, a handheld surgical robot with articulated end effectors has demonstrated advantages over conventional non-articulated endoscopic tools, offering a greater workspace, enhanced ergonomics, and improved performance in standard surgical tasks [34–36].

A primary focus within the area of patient support and follow-up is understanding the impact of pituitary surgery on QoL, demonstrated in the 1st, 5th, and 7th research priorities. Traditional assessments of surgical success typically rely on objective metrics such as the extent of resection, symptom improvement, and recurrence rates; however, these measures often overlook the patient’s subjective experience and overall QoL. To bridge this gap, patient-reported outcome measures (PROMs) can be used to more accurately capture the true impact of surgery on QoL. In response to this need, recent studies have developed PROMs specifically tailored for patients undergoing pituitary surgery [37]. While these measures hold promise, they require external validation and broader adoption to be effectively integrated into future research, ensuring that they address QoL concerns and align care with patient-centered priorities [38, 39]. Beyond QoL, addressing the psychological and social needs of patients undergoing pituitary surgery is equally critical, as reflected in the 5th and 7th research priorities. Meeting these needs is consistent with priorities set by other PSPs [40–42]. There are significant gaps in the provision of psychological, emotional, and social support for patients with pituitary adenomas, often exacerbated by the psychosocial burden placed on partners and caregivers [43, 44]. This highlights the urgent need for targeted research and the development of supportive interventions—including psychological, educational, and social resources—to effectively address the complex psychosocial challenges faced by patients, families, and caregivers throughout the treatment journey.

Accurately predicting postoperative complications and long-term outcomes remains a critical unmet need in pituitary surgery, as reflected in the 2nd, 9th, and 10th research priorities. Postoperative complications, such as CSF leak, which can occur in up to 5% of cases, reductions in QoL secondary to ongoing sinonasal issues, and panhypopituitarism, highlight the importance of addressing these challenges [8]. Effective prediction allows for improved risk stratification and the development of personalized treatment strategies, such as tailored monitoring, preventive measures, and optimized discharge protocols. Additionally, predicting remission or recurrence is essential for long-term management, particularly for NFPAs, where recurrence rates remain high—ranging from 30 to 50% within 5–10 years, even after radiologically confirmed complete resection [45–48]. Similar challenges exist with functioning adenomas, especially ACTH-secreting adenomas, where the risk of recurrence remains significant despite significant advancements in imaging and surgical techniques over the past four decades [10, 49]. To address these issues, emerging solutions, including multimodal machine learning tools and novel digital biomarkers, have been developed to enhance outcome prediction and support clinical decision-making [9]. These innovations aim to improve precision in post-operative risk assessments and guide follow-up strategies, potentially reducing recurrence rates and improving patient outcomes. While early evidence shows promise for these tools, further research is needed to validate their effectiveness and ensure their safe integration into clinical practice. Continued advancements in predictive technologies are crucial for bridging the gap in post-operative care and translating these developments into improved outcomes for patients undergoing pituitary surgery.

### Strengths and limitations

This study has several strengths. Importantly, the study comprised a diverse group of relevant stakeholders, including patients, charity representatives, and healthcare professionals, who provided valuable insights throughout the patient pathway and ensured that various treatment-related concerns were addressed. Furthermore, user-friendly outreach materials—such as posters, animated videos, articles, and presentations—were developed with support from public engagement specialists to promote awareness and facilitate participation (www.pit-cop.com). Strong engagement was evident throughout the process, with over 300 stakeholders contributing their perspectives, predominantly from patients. At each stage of the process, the PSP ensured balanced representation from all stakeholder groups. During the consensus meeting, there was equal representation of healthcare professionals and service users (1:1 ratio). Accordingly, these strengths assure that the final top 10 research priorities accurately reflect broad stakeholder involvement and a representative sample of the diverse perspectives and interests of all participants.

We acknowledge a number of limitations. The PSP was conducted via online surveys to identify and prioritize patient needs. This online approach may have inadvertently excluded individuals with challenged technical literacy and those with visual impairments in navigating the survey. Furthermore, despite considerable efforts to ensure survey dissemination, ethnic minority groups appeared to be underrepresented in the service user group, highlighting a limitation in capturing the perspectives and experiences of a more diverse population. Recognizing and addressing these barriers is crucial for fostering inclusivity, preventing health disparities, and ensuring that all individuals can voice their concerns and access the resources needed to improve their care.

### Implications and future research

The top 10 research priorities, available at www.pit-cop.com, offer researchers and funding agencies clear guidance on where to concentrate their efforts, both in the short and long term, while also informing decisions on resource allocation. We invite researchers and clinicians to collaborate on advancing the top 10 research priorities in pituitary surgery, as identified through our consensus process. The next steps involve translating these priorities into researchable questions and engaging with funders, patients, and healthcare professionals to design and deliver studies to address these important issues. It is important to recognize that all priorities discussed were considered of value. As such, our main summary report will also include the priorities not represented in the top 10, i.e., the 33 summary priorities and priorities 11–15 ranked at the consensus (Supplementary Table 3). This will be sent to our partner organisations including the Pituitary Society and the Pituitary Foundation to inform and guide any researchers focusing on these specific areas.

Future studies in tumor pathophysiology are required to better understand the interactions between adenoma tissue and adjacent structures, as well as the relationship between hormone production and cellular proliferation. These investigations will be essential for improving prognostic accuracy, supporting the development of targeted adjuvant therapies, and potentially uncovering new therapeutic targets for more personalized treatment strategies.

Assessing the impact of the PSP will be vital to ensure that identified priorities are being used to guide meaningful research and drive improvements in clinical practice. The impact of the PSP will be evaluated via an online survey distributed to our partner organizations following dissemination of the results. Respondents will be asked to describe any research grant applications, or any other research activity inspired by the PSP results. Furthermore, research outputs relating to the PSP will also be tracked via systematic reviews to identify studies addressing the priorities and to assess how frequently the top 10 list is cited in research articles. Additionally, we will identify changes in health policies, clinical guidelines, or practice standards that have integrated any of the PSP priorities. Finally, as new research addresses existing priorities, it will then be necessary to reevaluate and re-identify research priorities for pituitary surgery. This will ensure that research remains relevant and continues to reflect the current needs and interests of all stakeholder groups.

## Electronic supplementary material

Below is the link to the electronic supplementary material.

Supplementary file1 (DOCX 21 KB)

Supplementary file2 (DOCX 17 KB)

Supplementary file3 (DOCX 17 KB)

Supplementary file4 (MP4 49101 KB)

Supplementary file5 (MP4 317102 KB)

## Data Availability

Data is provided within the manuscript or supplementary information files.

## Appendix 1: Demographics of the pituitary surgery steering group members

**Table:**

Steering committee member	Stakeholder group/ speciality
Adam Mamelak	Neurosurgery
Alexandra Valetopoulou	Neurosurgery Trainee
Angelos Kolias	Neurosurgery
Ann McCormack	Endocrinology
Anouk Borg	Neurosurgery
Danyal Z Khan	Neurosurgery Trainee
Fion Bremner	Ophthalmology
Gabriel Zada	Neurosurgery
Gerald Raverot	Endocrinology
Hani Marcus	Neurosurgery
Inma Serrano	Clinical Nurse Specialist
Joy Ginn	Patient and Charity Representative
Juan Fernandez-Miranda	Neurosurgery
Katherine Miszkiel	Radiology
Maria Fleseriu	Endocrinology
Mark Gurnell	Endocrinology
Márta Korbonits	Endocrinology
Mathew Geltzeiler	Otolaryngology
Melmed Shlomo	Endocrinology
Michael Buchfelder	Neurosurgery
Michael Kosmin	Oncology
Neil Dorward	Neurosurgery
Nicola Newall	Neurosurgery Trainee
Olympia Koulouri	Endocrinology
Pat McBride	Patient and Charity Representative
Pierre Bouloux	Endocrinology
Richard Mannion	Neurosurgery
Simon Cudlip	Neurosurgery
Stephanie Baldeweg	Endocrinology
Steve Harris	Patient and Charity Representative
Theodore Schwartz	Neurosurgery
Tom Santarius	Neurosurgery
William Drake	Endocrinology
Zane Jaunmuktane	Pathology
