# Supplementary material for: Identifying research priorities for pituitary adenoma surgery: an international Delphi consensus statement
Source: Pituitary. 2025 Mar 5;28(2):36. doi: 10.1007/s11102-025-01502-7 (PMC11882698; doi:10.1007/s11102-025-01502-7)
Supplement: Supplementary file 1 — Supplementary file1 (DOCX 21 KB) [file 11102_2025_1502_MOESM1_ESM.docx]

**Supplementary Table 1**

Results of Interim Priority Setting Survey by Respondent group and Groups combined. Green – top 10 priority; Blue- priorities carried forward to final prioritisation workshop. ‘=’ indicates same rank.

| Priorities | Patients, carers and charity representatives (PCC) | | Healthcare professionals (HCP) | | Combined Group Ranking | |
| --- | --- | --- | --- | --- | --- | --- |
|  | **% PCC ranking Q in top 10** | **PCC rank** | **% HCPs ranking Q in top 10** | **HCP rank** | **% ranking in Top 10** | **Rank** |
| What is the impact of pituitary surgery on the long-term function and quality of life? | 79.7 | 1 | 21.1 | 22= | 52.9 | 3 |
| What are the causes of delayed diagnosis for patients with pituitary adenomas, and how can we address these factors to enhance prompt diagnosis and treatment? | 66.7 | 2 | 50.7 | 5 | 59.4 | 1= |
| What information and support do patients and their carers want and need throughout the patient journey? | 59.5 | 3 | 11.3 | 28= | 37.4 | 7= |
| How can we predict long-term outcomes, such as recurrence, after pituitary surgery? | 57.1 | 4= | 62.0 | 3= | 59.4 | 1= |
| How do pituitary adenomas affect mental health, and what is the best way to support patients? | 57.1 | 4= | 11.3 | 28= | 36.1 | 11= |
| What is the impact of pituitary adenomas on cognition and which treatments are effective in improving cognitive function? | 50 | 6 | 15.5 | 25= | 34.2 | 14= |
| Do genetic, environmental, or lifestyle factors contribute to the development of pituitary adenomas, and how can changing these factors help prevent or treat the condition? | 47.6 | 7 | 25.4 | 17= | 37.4 | 7= |
| How does surgical expertise, including the experience of the surgeon and the status of the pituitary centre, affect the management and outcomes of pituitary surgery? | 46.4 | 8 | 26.7 | 16 | 37.4 | 7= |
| How can we tailor decisions about management of pituitary adenomas to fit each individual patient's needs? | 41.7 | 9= | 29.6 | 14 | 36.1 | 11= |
| How can new surgical techniques and technologies, such as the use of robotics, improve outcomes after pituitary surgery? | 41.7 | 9= | 36.6 | 9 | 39.4 | 6 |
| Can pre-operative care, including specialist input, psychological support, support groups, and exercise, shorten hospital stays and improve outcomes for patients undergoing treatment for pituitary adenomas? | 40.5 | 11= | 11.3 | 28= | 27.1 | 20 |
| What is the optimal treatment for recurrent pituitary adenomas? | 40.5 | 11= | 31.0 | 13 | 36.1 | 11= |
| What is the optimal use of existing surgical techniques and technologies for different pituitary adenomas, such as giant adenomas, including transsphenoidal versus transcranial approaches, microscopic vs endoscopic techniques, and adjuncts such as image guidance? | 34.5 | 13 | 33.8 | 10= | 34.2 | 14= |
| Is there a role for screening for pituitary adenomas, and if so, when and how should this screening be conducted? | 29.8 | 14 | 12.7 | 27 | 22.0 | 24 |
| How can we predict early inpatient complications, such as dysnatraemia, after pituitary surgery, and can these be better managed or even prevented with empirical therapy? | 26.2 | 15= | 43.7 | 7 | 34.2 | 15 |
| What are the best ways to prevent or treat hypothalamic obesity associated with pituitary adenomas? | 26.3 | 15= | 25.4 | 17= | 25.8 | 22 |
| How do pituitary adenomas affect fertility, and how can fertility issues best be managed after surgery? | 25.0 | 17= | 21.1 | 22= | 23.2 | 23 |
| How can we optimise ophthalmic, biochemical, and imaging follow up for patients after pituitary surgery? | 25.0 | 17= | 32.4 | 12 | 28.4 | 19 |
| What is the ideal timing for surgical intervention for patients with different pituitary adenomas? | 22.6 | 19 | 46.5 | 6 | 33.5 | 17 |
| What visual parameters are crucial in the diagnosis, decision-making and prognostication of patients with pituitary adenomas? | 20.2 | 20= | 33.8 | 10= | 26.5 | 21 |
| How can we provide the best care for pituitary adenomas worldwide and what global health initiatives can we implement? | 20.2 | 20= | 18.3 | 24 | 19.4 | 25 |
| What are the clinical, biochemical, and imaging factors that can help determine the aggressiveness of pituitary adenomas? | 19.0 | 22= | 81.7 | 1 | 47.7 | 4 |
| What is the natural history of pituitary adenomas and how is it affected by surgery, medication, or radiotherapy (RT) treatment? | 19.0 | 22= | 40.8 | 8 | 37.4 | 7= |
| How can we best visualise pituitary adenoma and the surrounding structures on imaging, especially in cases not currently well seen, such as Cushing’s disease? | 15.5 | 24= | 62.0 | 3= | 36.8 | 10 |
| How can molecular profiling be used to improve the precision of pituitary adenoma management? | 15.5 | 24= | 76.1 | 2 | 43.2 | 5 |
| What are the best treatments for pain after pituitary surgery? | 14.3 | 26 | 9.9 | 31 | 12.3 | 30= |
| What is the socio-economic impact of pituitary surgery? | 13.1 | 27 | 7.0 | 32 | 10.3 | 32 |
| What are the treatment options for managing pituitary adenomas during pregnancy? | 11.9 | 28 | 15.5 | 25= | 13.5 | 29 |
| What is the optimal skull base reconstruction for patients undergoing pituitary surgery? | 8.3 | 29= | 28.2 | 15 | 17.4 | 26 |
| What are the best metrics for evaluating pituitary surgery? | 8.3 | 29= | 23.9 | 20= | 15.5 | 27= |
| What is the most cost-effective approach to the management of pituitary adenomas and its associated complications? | 7.1 | 31 | 25.4 | 17= | 15.5 | 27= |
| What cosmetic procedures are available to improve oral and facial features affected by acromegaly? | 6.0 | 32 | 5.6 | 33 | 5.8 | 33 |
| What is the optimal peri-operative anti-coagulation management in pituitary adenomas? | 3.6 | 33 | 23.9 | 20= | 12.9 | 30= |
