# Supplementary material for: Identifying research priorities for pituitary adenoma surgery: an international Delphi consensus statement
Source: Pituitary. 2025 Mar 5;28(2):36. doi: 10.1007/s11102-025-01502-7 (PMC11882698; doi:10.1007/s11102-025-01502-7)
Supplement: Supplementary file 2 — Supplementary file2 (DOCX 17 KB) [file 11102_2025_1502_MOESM2_ESM.docx]

**Supplementary Table 2**

Top 21 Research Priorities for Pituitary Surgery PSP: Original, Merged, and Non-Merged from the Consensus Workshop

| Top 21 Priorities Selected for Consensus Workshop |
| --- |
| What is the impact of pituitary surgery on the long-term function and quality of life? |
| What are the causes of delayed diagnosis for patients with pituitary adenomas, and how can we address these factors to enhance prompt diagnosis and treatment? |
| What information and support do patients and their carers want and need throughout the patient journey? |
| How can we predict long-term outcomes, such as recurrence, after pituitary surgery? |
| How do pituitary adenomas affect mental health, and what is the best way to support patients? |
| What is the impact of pituitary adenomas on cognition and which treatments are effective in improving cognitive function? |
| Do genetic, environmental, or lifestyle factors contribute to the development of pituitary adenomas, and how can changing these factors help prevent or treat the condition? |
| How does surgical expertise, including the experience of the surgeon and the status of the pituitary centre, affect the management and outcomes of pituitary surgery? |
| How can we tailor decisions about management of pituitary adenomas to fit each individual patient's needs? |
| How can new techniques and technologies, such as the use of robotics, improve outcomes after pituitary surgery? |
| What is the optimal treatment for recurrent pituitary adenomas? |
| What visual parameters are crucial in the diagnosis, decision-making and prognostication of patients with pituitary adenomas? |
| Can pre-operative care, including specialist input, psychological support, support groups, and exercise, shorten hospital stays and improve outcomes for patients undergoing treatment for pituitary adenomas? |
| What is the ideal sup for surgical intervention for patients with different pituitary adenomas? |
| What are the clinical, biochemical, and imaging factors that can help determine the aggressiveness of pituitary adenomas? |
| What is the optimal use of existing surgical techniques and technologies for different pituitary adenomas, such as giant adenomas, including transsphenoidal versus transcranial approaches, microscopic vs endoscopic techniques, and adjuncts such as image guidance? |
| How can we predict early inpatient complications, such as dysnatraemia, after pituitary surgery, and can these be better managed or even prevented with empirical therapy? |
| How can we optimise ophthalmic, biochemical, and imaging follow up for patients after pituitary surgery? |
| How can we best visualise pituitary adenoma and the surrounding structures on imaging, especially in cases not currently well seen, such as Cushing’s disease? |
| How can molecular profiling be used to improve the precision of pituitary adenoma management? |
| What is the natural history of pituitary adenomas and how is it affected by surgery, medication, or radiotherapy (RT) treatment? |
| Merged and Non-Merged Priorities from the Workshop |
| What is the impact of pituitary surgery on the long-term function and quality of life? |
| What are the causes of delayed diagnosis for patients with pituitary adenomas, and how can we address these factors to enhance prompt diagnosis and treatment? |
| "How can we predict long-term outcomes, such as recurrence, after pituitary surgery?  &How can molecular profiling be used to improve the precision of pituitary adenoma management?  & What are the clinical, biochemical, histological, and imaging factors that can help determine the aggressiveness of pituitary adenomas? " |
| "What information and support do patients and their carers want and need throughout the patient journey?  & Can pre-operative care, including specialist input, psychological support, support groups, and exercise, shorten hospital stays and improve outcomes for patients undergoing treatment for pituitary adenomas? " |
| "How can new surgical techniques and technologies, such as the use of robotics, improve outcomes after pituitary surgery?  & How can we best visualise pituitary adenoma and the surrounding structures on imaging, especially in cases not currently well seen, such as Cushing’s disease?" |
| How does surgical expertise, including the experience of the surgeon and the status of the pituitary centre, affect the management and outcomes of pituitary surgery? |
| "What is the natural history of pituitary adenomas and how is it affected by surgery, medication, or radiotherapy (RT) treatment?  & What visual parameters are crucial in the diagnosis, decision-making and prognostication of patients with pituitary adenomas?" |
| "How do pituitary adenomas affect mental health, and what is the best way to support patients? & What is the impact of pituitary adenomas on cognition and which treatments are effective in improving cognitive function? " |
| How can we predict early inpatient complications, such as dysnatraemia, after pituitary surgery, and can these be better managed or even prevented with empirical therapy? |
| How can we optimise ophthalmic, biochemical, and imaging follow up for patients after pituitary surgery? |
| Do genetic, environmental, or lifestyle factors contribute to the development of pituitary adenomas, and how can changing these factors help prevent or treat the condition? |
| How can we tailor decisions about management of pituitary adenomas to fit each individual patient's needs? |
| What is the ideal timing for surgical intervention for patients with different pituitary adenomas? |
| What is the optimal use of existing surgical techniques and technologies for different pituitary adenomas, such as giant adenomas, including transsphenoidal versus transcranial approaches, microscopic vs endoscopic techniques, and adjuncts such as image guidance? |
| What is the optimal treatment for recurrent pituitary adenomas? |
