# Supplementary material for: Identifying research priorities for pituitary adenoma surgery: an international Delphi consensus statement
Source: Pituitary. 2025 Mar 5;28(2):36. doi: 10.1007/s11102-025-01502-7 (PMC11882698; doi:10.1007/s11102-025-01502-7)
Supplement: Supplementary file 3 — Supplementary file3 (DOCX 17 KB) [file 11102_2025_1502_MOESM3_ESM.docx]

**Supplementary Table 3**

Research Priorities 11-15 for Pituitary Surgery PSP

**Rank Priorities 11-15**

| 11 | How can we tailor decisions about management of pituitary adenomas to fit each individual patient's needs? |
| --- | --- |
| 12 | What is the ideal timing for surgical intervention for patients with different pituitary adenomas? |
| 13 | What is the optimal use of existing surgical techniques and technologies for different pituitary adenomas, such as giant adenomas, including transsphenoidal versus transcranial approaches, microscopic vs endoscopic techniques, and adjuncts such as image guidance? |
| 14 | What is the optimal treatment for recurrent pituitary adenomas? |
| 15 | Do genetic, environmental, or lifestyle factors contribute to the development of pituitary adenomas, and how can changing these factors help prevent or treat the condition? |
